# Psychosocial predictors of health behavior adherence in heart-failure patients with comorbid depression: a secondary analysis of the Hopeful Heart trial

**DOI:** 10.1186/s40359-024-01816-4

**Published:** 2024-06-04

**Authors:** Alba Carrillo, Bea Herbeck Belnap, Scott D. Rothenberger, Robert Feldman, Bruce L. Rollman, Christopher M. Celano

**Affiliations:** 1 Instituto Polibienestar, University of Valencia, Valencia, Spain; 2 Department of Psychiatry, Massachusetts General Hospital, 125 Nashua Street, Suite 324, Boston, MA 02114 USA; 3 Harvard Medical School, Boston, MA USA; 4 Division of General Internal Medicine, Center for Research on Health Care, University of Pittsburgh School of Medicine, Pittsburgh, PA USA; 5 Center for Behavioral Health, Media, and Technology, University of Pittsburgh School of Medicine, Pittsburgh, PA USA; 6 Division of General Internal Medicine, Center for Research on Health Care Data Center, University of Pittsburgh School of Medicine, Pittsburgh, PA USA

**Keywords:** Depression, Heart failure, Health behavior, Adherence

## Abstract

**Background:**

Depression affects 20–30% of individuals with heart failure (HF), and it is associated with worse health outcomes independent of disease severity. One potential explanation is the adverse impact of depression on HF patients’ adherence to the health behaviors needed to self-manage their condition. The aim of this study is to identify characteristics associated with lower adherence in this population, which could help to recognize individuals at higher risk and eventually tailor health behavior interventions to their needs.

**Methods:**

Using data from a randomized, controlled, collaborative care treatment trial in 629 patients with HF and comorbid depression, we performed mixed effects logistic regression analyses to examine the cross-sectional and prospective relationships between medical and psychosocial variables and health behavior adherence, including adherence to medications, a low-sodium diet, and physician appointments.

**Results:**

In cross-sectional analyses, married marital status and higher physical health-related quality of life (HRQoL) were associated with greater overall adherence (compared to married, single Odds Ratio [OR] = 0.46, 95% Confidence Interval [CI] = 0.26–0.80; other OR = 0.60, CI = 0.38–0.94; *p* = .012. Physical HRQoL OR = 1.02, CI = 1.00-1.04, *p* = .047). Prospectively, greater levels of social support were associated with improved overall adherence one year later (OR = 1.04, 95% CI = 1.00-1.08, *p* = .037). Social support, HF symptom severity, race and ethnicity, and age were predictors of specific types of adherence. Neither depression nor optimism was significantly associated with adherence outcomes.

**Conclusions:**

These results provide important preliminary information about risk factors for poor adherence in patients with both HF and depression, which could, in turn, contribute to the development of interventions to promote adherence in this high-risk population.

**Trial registration:**

https://clinicaltrials.gov/ct2/show/NCT02044211; registered 1/21/2014.

## Introduction

Heart failure (HF) affects more than 23 million people worldwide and is associated with impaired quality of life, reduced functional capacity, and high rates of mortality [1]. Among individuals with HF, and particularly those with reduced ejection fraction, adherence to health behaviors, such as taking medications regularly and adhering to a low-sodium diet, is associated with a reduced risk of HF exacerbations, hospitalizations, and mortality [1–3]. However, many HF patients struggle to adhere to these behaviors [4, 5].

Psychological health plays an important role in adherence in patients with HF. Depression affects 20–30% of HF patients [6, 7]. Higher levels of depression have been associated with poorer adherence to HF self-care [3, 4, 8], as well as increased risks of recurrent cardiac events, hospitalizations, and mortality [7, 9]. In fact, depression treatment has been proposed as a strategy to improve self-care behaviors for HF, as it might help with depressive symptoms that could interfere with engagement in these behaviors (i.e., fatigue, lack of concentration or hopelessness) [10, 11]. There are effective treatment strategies for depression in this population [7], though further research is needed to determine the effects of these programs on self-care behaviors [10]. In contrast, the presence of positive psychological constructs such as optimism, self-efficacy, and perceived social support are linked to higher adherence to self-care behaviors (e.g., dietary adherence, symptom monitoring), as well as better health outcomes and fewer cardiac-related events [12–17].

Sociodemographic and medical factors also have the potential to influence adherence to health behaviors among patients with HF. Observational studies have found that White HF patients show greater health behavior adherence than individuals from racial and ethnic minority backgrounds [18, 19]. Additionally, though results are mixed, male and older HF patients appear to be more adherent to care (e.g., medication adherence, cardiac rehabilitation) [20–22]. Other sociodemographic variables such as higher levels of formal education, income, and socioeconomic status have been linked to higher health-behavior adherence in chronic conditions [3, 23, 24]. Regarding medical factors, individuals with more HF symptoms and those with certain comorbidities (e.g., pulmonary, renal disease) appear to be more likely to engage in self-care activities or interventions to improve self-care [3, 25, 26]. Finally, the relationships between self-care and health-related quality of life (HRQoL) in patients with HF is mixed, with some studies finding that self-care interventions improve HRQoL and others finding no effects of self-care interventions on HRQoL [27]. Despite the established links between psychological, sociodemographic, and medical factors and adherence in HF patients, little is known about those relationships in individuals with comorbid HF *and* depression. Given the inverse relationship between depression and adherence in this population, a better understanding of the factors associated with poor health behavior adherence is critical. This may help to identify patients at highest risk for poor medical outcomes and could inform the development of treatments that target those specific populations.

Accordingly, we examined the cross-sectional and prospective relationships between psychological, sociodemographic, and medical variables and health behavior adherence in 629 patients with HF and comorbid depression who participated in the *Hopeful Heart* trial, a randomized controlled trial of a multicomponent collaborative care intervention [28, 29]. We hypothesized that depressive symptom severity would predict lower levels of adherence to health behaviors in this sample, and that optimism, social support, and HRQoL would be positively associated with adherence. We also explored the relationships between other sociodemographic and medical variables and adherence.

## Methods

We performed secondary analyses of data from the *Hopeful Heart trial*, a randomized controlled trial to examine the efficacy of a 12-month, telephone-delivered collaborative care intervention for patients with HF and comorbid major depressive disorder. Full details about the design and conduct of the *Hopeful Heart* trial have been published elsewhere [28, 29]. This study was performed in line with the principles of the Declaration of Helsinki. All participants provided written informed consent prior to participation, and the University of Pittsburgh Institutional Review Board approved the study prior to initiation of recruitment. We registered the study in ClinicalTrials.gov (NCT02044211; registered 1/21/2014) before the start of enrollment.

### Participants

We screened hospitalized adults (> 21 years) with a diagnosis of HF, left ventricular ejection fraction of ≤ 45%, and New York Heart Association (NYHA) class II, III, or IV symptoms, for depression using the 2-item Patient Health Questionnaire (PHQ-2). Two weeks after hospital discharge study assessors contacted patients who screened positive [30] via telephone to confirm a moderate level of depression, defined as ≥ 10 on the 9-item Patient Health Questionnaire (PHQ-9) [31]. We excluded patients if they were experiencing cognitive impairment, an unstable non-cardiovascular medical condition with less than one year life expectancy, a psychotic illness, if they were in active treatment for depression or anxiety, or if they did not have household telephone or could not communicate in English.

### Procedures

We randomized participants with evidence of current depressive symptoms (PHQ 9 ≥ 10; *N* = 629) either to a “blended” collaborative care intervention for both HF and depression (“blended”), a collaborative care intervention for HF only (enhanced usual care, “eUC”), or their physicians’ usual care (“UC”) for HF and depression. As part of the collaborative care strategy, participants in the blended and eUC groups received regular phone calls (at least twice per month for 3–4 months, then monthly thereafter) from trained study nurse care managers over a 12-month period; both groups received support for cardiovascular symptoms, while the blended collaborative care group additionally received treatment recommendations for depression. At baseline and at a 12-month follow-up timepoint, blinded assessors administered telephone assessments.

### Measures

#### Psychological measures and HRQoL

Measures related to psychological health and HRQoL were obtained at baseline and 12-month follow-up. Psychological measures included social support, optimism, and depression [33]. We assessed social support with the ENRICHD Social Support Inventory (ESSI [32]), . We used the Life Orientation Test-Revised (LOT-R [33]), to measure optimism and the nine-item Patient Health Questionnaire-9 (PHQ-9 [31]), to measure depression. We assessed physical HRQoL with the Physical Component Summary of the Short-Form 12 Health Survey (SF-12 PCS [34]). More information about the measures can be found in Table 1.

#### Sociodemographic and medical measures

At baseline, participants self-reported their age, gender, race and ethnicity, marital status, education level, employment status, and NYHA class. Study staff supplemented this information with detailed reviews of participants’ health records for information related to medical conditions pertinent to HF (e.g., lowest documented ejection fraction, hyperlipidemia, hypertension, history of myocardial infarction).

#### Measures of adherence

To measure medication adherence and adherence to a low-sodium diet, we adapted our questions from prior work in individuals with heart disease [35]. Specifically, to assess medication adherence in the preceding two weeks, we asked participants to rate on a 5-point Likert scale (1: “never/rarely;” 5: “all of the time”) how frequently they had difficulty remembering to take their cardiac medications as prescribed. Similarly, to assess adherence to a low-sodium diet, participants rated their frequency of sodium intake (“How often did you eat salty food?”) over the past two weeks on a 5-point Likert scale (1: “never”, 5: “every day of the week”). We considered patients adherent to medication or low-sodium diet if they responded “never/rarely” or “once a week” to these questions. Additionally, we measured adherence to physician appointments over the previous two weeks with a dichotomous (yes/no) question (“Have you missed a scheduled doctor’s appointment?”). Finally, we calculated an overall measure of adherence to health behaviors, including the aforementioned items. We considered patients adherent (“adherers”) when they responded “never/rarely” or “once a week” to both the medication and the low-sodium diet questions, and “no” to the question about adherence to physician appointments. These measures were assessed at baseline and at 12-month follow-up.

### Data analysis

We conducted statistical analyses using R (version 3.6.0). To examine differences between adherers and non-adherers in their sociodemographic, psychological, and medical characteristics, we performed ANOVA F-tests for continuous variables and chi-square tests for categorical variables. We performed mixed effects logistic regression analyses to assess cross-sectional and prospective relationships between baseline psychological, sociodemographic, and medical variables and overall health behavior adherence at baseline and 12 months later. We then performed the same analyses for each adherence outcome measure individually (medication adherence, attendance at physician appointments, and adherence to a low-sodium diet). We included fixed effects for age, gender, race and ethnicity, marital status, NYHA class, hyperlipidemia, PCS, ESSI, LOT-R, PHQ-9, and treatment arm; a binary time variable indicating whether an observation is at baseline or 12-month follow up; and two-way interactions between each covariate and the time indicator. Additionally, we included a random intercept for participant to account for repeated measures and participant-level variability at baseline. Odds ratios corresponding to baseline adherence were estimated from main effects in the model, and odds ratios corresponding to follow-up adherence were estimated from linear combinations of main and interaction effects. Models were fit via maximum likelihood estimation under the assumption that missing data were missing at random. All tests were two-tailed with a significance level α = 0.05, and no adjustments were made for multiplicity.

## Results

### Baseline characteristics

A total of 629 participants were included in our analyses. In terms of race and ethnicity, participants were divided into two groups: White (75.2%) and Non-white (including “African-American” 22.9%, and “other” 1.9%). In unadjusted analyses (Table 1) at baseline, compared to non-adherers, adherers tended to be older (65.2 vs. 61.8 years, *p* = .001), White (78.9% vs. 70.7%, *p* = .017), and married (52.6% vs. 36.7%, *p* < .001), and they were more likely to have hyperlipidemia (75.7% vs. 68.2%, *p* = .036), higher levels of social support (ESSI: 27.2 vs. 25.3, *p* < .001), and higher levels of optimism (LOT-R: 19.1 vs. 18.0, *p* = .005). On average, participants reported moderate depressive symptoms overall (mean PHQ-9 for the total sample = 14.08 [SD 3.6]), and there were no significant differences in depressive symptoms between adherers and non-adherers (PHQ-9: 13.88 vs. 14.31, *p* = .14). There were no significant between-group differences in the remaining sociodemographic, psychological, and medical variables (see Table 1).

**Table 1:** Sociodemographic, psychological, and medical baseline characteristics between adherers and non-adherers

Measure* n (%)	Total (*n* = 629)	Adherers (*n* = 346)	Non-adherers (*n* = 283)	Test statistic^†^, *p*-value
Age	63.63 ± 12.93	65.17 ± 12.42	61.75 ± 13.31	*F =* 11.073, *p* = .001
Gender (*female*)	273 (43.4%)	154 (44.5%)	119 (42%)	χ^2^ (1) = 0.383, *p* = .54
Race and ethnicity				
*White*	473 (75.2%)	273 (78.9%)	200 (70.7%)	χ^2^ (1) = 5.654, *p* = .017
*Non-white* ^*1*^	156 (24.8%)	73 (21.1%)	83 (29.3%)	
Education (*≥* *College)*	322 (51.2%)	172 (49.7%)	150 (53%)	χ^2^ (1) = 0.68, *p* = .41
Marital status				
*Married* ^*2*^	286 (45.5%)	182 (52.6%)	104 (36.7%)	χ^2^ (2) = 18.20, *p* < .001
*Single*	121 (19.2%)	51 (14.7%)	70 (24.7%)	
*Other* ^*3*^	222 (35.3%)	113 (32.7%)	109 (38.5%)	
Employment (*working*)	75 (12%)	39 (11.3%)	36 (12.7%)	χ^2^ (1) = 0.28, *p* = .60
SF-12 PCS *(mean ± sd)* Range: 0-100	29.36 ± 9.7	30.02 ± 10.02	28.55 ± 9.25	*F =* 3.56, *p* = .060
ESSI *(mean ± sd)* Range: 8–34	26.34 ± 6.34	27.16 ± 6.01	25.34 ± 6.59	*F =* 13.09, *p* < .001
LOT-R *(mean ± sd)* Range: 0–24	18.58 ± 4.59	19.05 ± 4.6	18.01 ± 4.52	*F =* 8.08, *p* = .005
PHQ-9 *(mean ± sd)* Range: 0–27	14.08 ± 3.6	13.88 ± 3.7	14.31 ± 3.48	*F =* 2.19, *p* = .14
Group				
*UC*	126 (20%)	78 (22.5%)	48 (17%)	χ^2^ (2) = 3.19, *p* = .20
*Blended*	251 (39.9%)	136 (39.3%)	115 (40.6%)	
*eUC*	252 (40.1%)	132 (38.2%)	120 (42.4%)	
NYHA Class				
*II*	213 (33.9%)	104 (30.1%)	109 (38.5%)	χ^2^ (2) = 4.98, *p* = .083
*III*	341 (54.2%)	198 (57.2%)	143 (50.5%)	
*IV*	75 (11.9%)	44 (12.7%)	31 (11%)	
LEF *(mean ± sd)*	28.25 ± 9.3	28.61 ± 9.42	27.81 ± 9.14	*F =* 1.16, *p* = .28
Hypertension	540 (86%)	298 (86.1%)	242 (85.8%)	χ^2^ (1) = 0.01, *p* = .91
Diabetes	329 (52.4%)	170 (49.1%)	159 (56.4%)	χ^2^ (1) = 3.27, *p* = .070
Hyperlipidemia	455 (72.3%)	262 (75.7%)	193 (68.2%)	χ^2^ (1) = 4.41, *p* = .036
Myocardial infarction	285 (45.4%)	164 (47.4%)	121 (42.9%)	χ^2^ (1) = 1.26, *p* = .26
CABG	177 (28.2%)	98 (28.4%)	79 (27.9%)	χ^2^ (1) = 0.02, *p* = .89
ICD	223 (35.6%)	118 (34.3%)	105 (37.2%)	χ^2^ (1) = 0.58, *p* = .45

Notes*All measures present data on n (%) unless otherwise specified. For SF-12 PCS, ESSI and LOT-R, higher scores indicate greater physical health, social support and optimism, respectively; while for PHQ-9, higher scores indicate more severe depression. †All F-tests had 1 numerator df and 627 denominator df. Degrees of freedom for chi-square tests are shown in parentheses. ^*1*^Non-white including “African-American” (22.9%) and “other” (1.9%), ^*2*^Married or living with one’s partner, ^*3*^Separated/divorced/widowed/other, Refused or Missing. CABG. = Coronary Artery Bypass Grafting. ESSI = ENRICHD Social Support Inventory. eUC = Enhanced Usual Care. ICD = Implantable Cardiac Defibrillator. LEF. = Lowest ejection fraction. LOT-R = Life Orientation Test-Revised. NYHA Class = New York Heart Association Functional Classification. PHQ-9 = Patient Health Questionnaire-9. SF-12 PCS = Physical Component Summary of the Short-Form 12 Health Survey. UC = Usual Care

### Cross-sectional relationships between psychological, medical, and sociodemographic factors and adherence

In mixed effects logistic regression analyses, marital status and physical HRQoL were associated with reported health behavior adherence at baseline. More specifically, unmarried individuals and those who did not live with a partner were less likely to be adherent to health behavior recommendations compared to married individuals (single: Odds Ratio [OR] = 0.46, 95% Confidence Interval [CI] = 0.26–0.80; other: OR = 0.60, CI = 0.38–0.94; *p* = .012). In addition, patients with greater physical HRQoL were more likely to be adherent to health recommendations (OR = 1.02, CI = 1.00-1.04, *p* = .047; see Table 2 for detailed results).

Sociodemographic and medical factors were also associated with specific types of reported adherence. Compared to White individuals, those from racial and ethnic minority backgrounds were less likely to adhere to prescribed medications or to attend physician appointments (medications: OR = 0.36, CI = 0.15–0.86, *p* = .022; appointments: OR = 0.51, CI = 0.30–0.88, *p* = .015). Conversely, individuals with more severe HF symptoms (i.e., higher NYHA class) were more likely to report adherence to prescribed medications (compared to NYHA class II; NYHA class III OR = 2.50, CI = 1.08–5.78; NYHA class IV, OR = 7.31, CI = 0.89–60.09; *p* = .036). No other sociodemographic, medical, or psychological variables were associated with adherence at baseline.

**Table 2:** Cross-sectional relationships between psychosocial factors and adherence (overall adherence, medication adherence, physician follow up, and low-sodium diet adherence) at baseline (*N* = 629)

Variables	Overall adherence OR (95% CI), *p*-value	Medication intake OR (95% CI), *p*-value	Physician attendance OR (95% CI), *p*-value	Low-sodium diet OR (95% CI), *p*-value
Age	1.015 (0.998–1.032), *p =* .09	1.003 (0.969–1.039), *p =* .86	0.997 (0.977–1.017), *p =* .73	1.016 (1.000-1.033), *p =* .06
Gender (*ref.: male*)				
*Female*	1.258 (0.849–1.862), *p =* .25	1.146 (0.491–2.675), *p =* .75	1.350 (0.832–2.190), *p =* .22	1.002 (0.679–1.479), *p =* .99
Race and ethnicity (*ref.: white*)				
*Non-white*	0.858 (0.533–1.381), *p =* .53	0.358 (0.149–0.859), *p =* .022*	0.514 (0.301–0.877), *p =* .015*	1.040 (0.652–1.661), *p =* .87
Marital Status (*ref.: married*)	*p =* .012*	*p =* .68	*p* = .41	*p =* .31
*Single*	0.460 (0.264-0.800)	0.616 (0.207–1.837)	0.658 (0.345–1.257)	0.703 (0.410–1.204)
*Other*	0.597 (0.378–0.943)	0.786 (0.288–2.143)	0.757 (0.428–1.340)	0.741 (0.471–1.165)
NYHA class (*ref.: II*)	*p =* .10	*p =* .036*	*p =* .42	*p =* .47
*III*	1.461 (0.962–2.221)	2.501 (1.082–5.777)	1.405 (0.847–2.328)	1.099 (0.727–1.662)
*IV*	1.833 (0.965–3.485)	7.310 (0.889–60.085)	1.194 (0.562–2.536)	1.501 (0.784–2.872)
Hyperlipidemia (*ref.: yes*)				
*No*	0.718 (0.465–1.108), *p =* .13	2.557 (0.868–7.530), *p =* .09	0.665 (0.401–1.104), *p =* .11	0.807 (0.527–1.236), *p =* .32
SF-12 PCS	1.020 (1.000-1.041), *p =* .047*	1.022 (0.978–1.067), *p =* .34	1.022 (0.996–1.048), *p =* .09	1.007 (0.987–1.027), *p =* .51
ESSI	1.029 (0.995–1.063), *p =* .09	1.017 (0.954–1.084), *p =* .60	1.018 (0.980–1.059), *p =* .36	1.029 (0.997–1.063), *p =* .08
LOT-R	1.033 (0.987–1.082), *p =* .16	0.984 (0.893–1.084), *p =* .74	1.018 (0.962–1.077), *p =* .53	1.018 (0.973–1.065), *p =* .44
PHQ-9	0.984 (0.932–1.038), *p =* .54	0.919 (0.827–1.021), *p =* .11	0.974 (0.914–1.039), *p =* .42	0.989 (0.938–1.043), *p =* .68

*Notes* ESSI = ENRICHD Social Support Inventory. LOT-R = Life Orientation Test-Revised. PHQ-9 = Patient Health Questionnaire-9. SF-12 PCS = Physical Component Summary of the Short-Form 12 Health Survey. * = *p* < .05

### Prospective relationships between psychological, sociodemographic, and medical factors and adherence

The only prospective predictor of overall reported adherence at 12-months was social support (see Table 3): greater levels of social support were associated with greater overall adherence (OR = 1.04, CI = 1.00-1.08, *p* = .037). This finding was driven by higher rates of attendance at physician follow-up appointments (OR = 1.05, CI = 1.00-1.10, *p* = .034) and higher adherence to a low-sodium diet (OR = 1.04, CI = 1.00-1.08, *p* = .047). Finally, age was associated with greater adherence to prescribed medications and physician follow-up at 12-months. Specifically, with each one-year increase in age, participants had 4% increased odds of remembering to take their medications as prescribed (OR = 1.04, CI = 1.00-1.09, *p* = .043) and 3% increased odds of attending scheduled physician appointments (OR = 1.03, CI = 1.01–1.06, *p* = .014).

**Table 3:** Prospective relationships between psychosocial factors at baseline and adherence (overall adherence, medication adherence, physician follow up, and low-sodium diet adherence) at follow-up (*N* = 629)

Variables	Overall adherence OR (95% CI), *p*-value	Medication intake OR (95% CI), *p*-value	Physician follow-up OR (95% CI), *p*-value	Low-sodium diet OR (95% CI), *p*-value
Age	1.015 (0.996–1.034), *p =* .12	1.043 (1.001–1.087), *p =* .043*	1.032 (1.006–1.057), *p =* .014*	1.007 (0.989–1.025), *p =* .45
Gender (*ref.: male*)				
*Female*	0.861 (0.550–1.349), *p =* .51	1.000 (0.374–2.673), *p >* .99	0.749 (0.423–1.328), *p =* .32	1.091 (0.706–1.687), *p* = .69
Race and ethnicity (*ref.: white*)				
*Non-white*	0.810 (0.472–1.390), *p =* .44	0.423 (0.144–1.243), *p* = .12	0.802 (0.415–1.552), *p =* .51	0.764 (0.453–1.288), *p =* .31
Marital Status (*ref.: married*)	*p =* .73	*p =* .42	*p =* .32	*p =* .70
*Single*	1.273 (0.675–2.398)	0.559 (0.175–1.785)	1.808 (0.772–4.236)	1.210 (0.658–2.225)
*Other*	1.151 (0.687–1.928)	1.178 (0.327–4.250)	0.989 (0.517–1.890)	1.218 (0.737–2.012)
NYHA class (*ref.: II*)	*p =* .49	*p =* .56	*p =* .36	*p =* .22
*III*	0.855 (0.533–1.373)	0.632 (0.215–1.861)	0.786 (0.413–1.493)	0.822 (0.519–1.302)
*IV*	1.274 (0.619–2.622)	1.299 (0.215–7.861)	0.536 (0.228–1.259)	1.473 (0.721–3.010)
Hyperlipidemia (*ref.: yes*)				
*No*	0.804 (0.488–1.326), *p = .*39	1.551 (0.507–4.748), *p =* .44	1.283 (0.669–2.459), *p =* .45	0.748 (0.462–1.210), *p =* .24
SF-12 PCS	0.996 (0.973–1.019), *p =* .70	0.965 (0.918–1.014), *p =* .15	1.030 (0.998–1.062), *p =* .07	0.990 (0.968–1.012), *p =* .37
ESSI	1.041 (1.003–1.080), *p =* .037*	1.013 (0.940–1.090), *p =* .74	1.051 (1.004-1.100), *p =* .034*	1.037 (1.000-1.075), *p =* .047*
LOT-R	1.018 (0.968–1.071), *p =* .49	0.981 (0.881–1.093), *p =* .73	0.987 (0.925–1.052), *p =* .68	1.034 (0.985–1.086), *p =* .18
PHQ-9	1.018 (0.958–1.082), *p =* .56	0.906 (0.805–1.019), *p =* .10	1.009 (0.934–1.090), *p =* .82	1.052 (0.991–1.116), *p =* .10
Treatment (*ref.: usual care*)	*p* = .75	*p* = .63	*p* = .24	*p* = .60
*Blended care*	1.192 (0.659–2.157)	1.048 (0.313–3.507)	0.725 (0.312–1.681)	1.320 (0.743–2.344)
*Enhanced usual care*	1.014 (0.561–1.834)	1.689 (0.451–6.323)	0.516 (0.227–1.173)	1.305 (0.736–2.312)

*Notes* ESSI = ENRICHD Social Support Inventory. LOT-R = Life Orientation Test-Revised. PHQ-9 = Patient Health Questionnaire-9. SF-12 PCS = Physical Component Summary of the Short-Form 12 Health Survey. * *p* < .05

## Discussion

In a large sample of individuals with HF and comorbid depression, we identified several important factors associated with self-reported health behavior adherence. In cross-sectional analyses, marital status (being married or living with a partner) and higher physical HRQoL were associated with greater overall adherence. Furthermore, higher NYHA class was linked to better medication adherence, and reported White race and ethnicity was associated with both better medication adherence and attendance at physician appointments.

In prospective analyses, higher perceived social support was associated with greater self-reported overall adherence, with adherence to follow-up physician appointments, and to a low-sodium diet 12 months later. Older age was a significant predictor of greater adherence to prescribed medications and physician follow-up appointments one year later. We did not find other significant relationships between psychological variables (optimism, depression) and health behavior adherence.

In terms of the psychological measures (and among all included variables), social support was the measure most consistently associated with adherence in this sample of depressed HF patients. Living with a partner or being married was cross-sectionally associated with greater overall health behavior self-reported adherence, and self-reported social support predicted overall adherence and attending to doctor’s appointments and following a low-sodium diet at the 12-month assessment. These findings are in line with previous studies: social support has been linked to greater adherence to health behaviors in HF patients [14, 15]. Relatives and partners can assist patients with their treatment regimens, provide guidance on symptom monitoring, or promote healthy behaviors such as following a low-sodium diet or taking prescribed medication, through encouragement, provision of reminders and emotional support, or monitoring their HF symptoms [15]. Other authors suggest that social support might contribute to patients’ adherence by increasing their self-care confidence, that is, enhancing their confidence in their ability to perform and stick to health behaviors [36]. Therefore, the impact of social support on adherence can have important clinical consequences, given the association between engagement in health behaviors and improved health outcomes [5, 37].

Contrary to our hypotheses, neither depressive symptoms nor optimism were associated with any adherence outcomes, either cross-sectionally or prospectively. These findings differ from several prior studies that have established independent relationships between optimism and adherence to health behaviors (such as physical activity and a low sodium diet) in HF populations [4, 7, 38]. Results differ, as well, from a wide body of research that has found an association between the presence of depressive symptoms and non-adherence in cardiac patients [7, 39]. However, in contrast to prior studies, all participants in this study were depressed. The relationships between psychological constructs and health behavior adherence may be different in this population compared to the general population of individuals with HF. Furthermore, the current study did not measure physical activity, one of the health behaviors most clearly associated with depressive symptoms and optimism in cardiac patients [7, 38]. These may help explain the discrepancy in the findings between the current and previous studies.

Our findings related to race and ethnicity and age extend those of prior studies. The finding of lower rates of adherence to health behaviors among individuals from racial or ethnic minority backgrounds (compared to their White counterparts) is consistent with previous works and may be related to different cultural backgrounds, health-care related costs, socioeconomic status (SES), historical disadvantages, and mistrust of the healthcare system [1, 40–43]. However, these results were not confirmed in the prospective analyses, highlighting the need for further research in this area. Our finding that age was associated with both medication adherence and attendance at physician appointments is consistent with some prior work [3, 21], though results from previous studies and systematic reviews on this matter are mixed [21, 44]. Given the mixed results of prior studies, additional work should be done to clarify the relationship between age and adherence among HF patients, particularly those with depression.

Medical status also appears to play a role in adherence in depressed HF patients. Individuals with more severe HF symptoms were more likely to take medications regularly. Although this relationship seems to be under-explored, these results are in line with a previous study [26], which found that education level and symptom severity were associated with self-care. One hypothesis for this finding is that patients with greater symptom burden may be more aware of the benefits of medication on their symptoms. Additionally, those patients with better physical HRQoL showed higher overall adherence rates at baseline. To our knowledge, there are no previous studies examining physical HRQoL as a predictor of adherence to medication intake, low sodium diet and/or physician appointments. In contrast, a number of studies have examined the impact of self-care interventions on HRQoL, and the results of these studies have been mixed [27]. We would hypothesize that patients with higher HRQoL might find fewer physical barriers to follow recommended health behaviors, which might facilitate their adherence. Conversely, it could also be possible that individuals with greater levels of adherence derive HRQoL-related benefits as a result of this adherence.

This study has several limitations. The measures of adherence to health behaviors were self-reported and based on single-item questions. Furthermore, the findings of this study pertain to a specific group of HF patients (i.e., English-speaking adults with reduced ejection fraction, comorbid depression, and access to a telephone); therefore, results may not be generalizable to other groups of HF patients. In addition, we did not assess the impact of financial factors (e.g., medication costs, co-pays at physician appointments) and participant SES on adherence, despite evidence that these factors are associated with nonadherence [45–47]. This has the potential to affect our findings related to race and ethnicity, as racial or ethnic background and SES are related to each other [43]. Finally, we did not examine the relationships between adherence measures and clinical outcomes such as hospital readmissions, recovery from depression, or mortality. Future studies that include validated scales or objective measures of adherence (e.g., claims data for physician appointments, laboratory markers of sodium intake), enroll a broader group of patients with HF (e.g., with reduced and preserved ejection fraction, with different depression levels, and from more diverse racial and ethnic backgrounds), include measures of SES, and examine the relationships between adherence and medical outcomes would help to confirm the results obtained in this work.

Despite these limitations, results from this study provide important preliminary information about factors that may contribute to non-adherence in patients with both depression and HF. Though further study is needed to confirm these findings, they suggest that it may be useful to identify individuals at elevated risk for non-adherence to such behaviors, namely younger individuals, those from racial or ethnic minority backgrounds, and those with low levels of social support, lower physical health-related quality of life, and less severe HF symptoms. Further research is needed to determine how to approach those individuals at higher risk of non-adherence most effectively. Additionally, these results could be used in the development of interventions—or modification of available interventions—that target those factors most strongly associated with adherence (e.g., social support). Given that the Hopeful Heart blended CC program did not significantly impact adherence, we also would consider adjusting the intervention to focus more specifically on those factors (e.g., social support) that are most clearly linked with adherence. Ultimately, if these interventions significantly impact health behavior adherence, they have the potential to improve health outcomes in individuals with heart failure and comorbid depression.

## Data Availability

The datasets used and/or analyzed during the current study are available from the corresponding author on reasonable request.
